# A Finger on the Chin: Rare Report of a Faciomandibular Teratoma in a Nepalese Infant

**DOI:** 10.1155/crpe/9921032

**Published:** 2025-06-16

**Authors:** Ashish Lal Shrestha, Dakshata Shakya, Sagar Khadka, Pranita Gurung

**Affiliations:** ^1^Department of Pediatric Surgery, Kathmandu Medical College and Teaching Hospital, Sinamangal, P.O. Box 12127, Kathmandu, Nepal; ^2^Department of Radiodiagnosis, Kathmandu Medical College and Teaching Hospital, Sinamangal, P.O. Box 12127, Kathmandu, Nepal; ^3^Department of Pathology, Kathmandu Medical College and Teaching Hospital, Sinamangal, P.O. Box 12127, Kathmandu, Nepal

## Abstract

**Background:** Teratoma is a tumor usually consisting of tissues derived from multiple germ layers. A congenital teratoma occurring in the region of the head and neck is rare with barely 10 reports in the global literature since 1996. Of further rarity is its mandibular location. This probably represents the first report of its kind in the world in addition to being the first one to be reported from Nepal.

**Case Presentation:** We report a case of an 11-month-old infant with a congenital appendage over the side of the chin that was treated with surgical excision and histologically confirmed as mature teratoma.

**Conclusion:** Congenital facial teratoma in the mandibular location is a rare event. A principle combining appropriate surgical technique and correct oncological principles keeping esthetic appearance in mind is necessary for the best outcome.

## 1. Introduction

Teratomas are neoplasms that comprise several tissues that are not native to the area of origin. These are derived from the primordial germ cells and usually composed of tissues from the three embryonic germ layers, namely, ectoderm, mesoderm, and endoderm [1]. The most frequently observed sites are the sacro-coccyx, testicles, ovaries, anterior mediastinum, and retroperitoneum.

The head and neck, however, is a rare location, with teratomas here accounting for only 0.47%–6% of all the cases with a reported incidence of 2.5–5/100,000 among live births [2]. Again, of these, lateral facial teratomas are even rarer and usually associated with neurological and bony deformities. Complete surgical excision is usually curative supplemented with regular follow-up to detect early recurrences. Hereby, we present a case of mature facial teratoma.

## 2. Case Report

**Figure 1:**
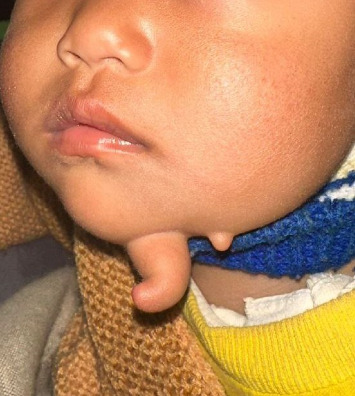
A finger-like fleshy lesion over the left side of the chin with normal overlying skin and an adjacent satellite lesion.

An 11-month-old infant girl was evaluated for a projecting lesion over the chin that closely resembled a finger. Although, noted soon after birth, lacking symptoms, it remained largely unaddressed until a gradual progress was observed to the current size. General physical examination was unremarkable while local examination showed a 4 × 1-cm finger-like swelling over the left side of the chin with a smooth surface and a normal overlying skin. It was soft in consistency except at the base where a firm structure was palpable. It was noncompressible and did not have any signs of inflammation. Just lateral to it, another 0.5 × 0.5-cm satellite lesion was noted resembling a skin tag, as shown in Figure 1.

No other lesions were noted in the head and neck, neither any facial deformities nor palpable regional lymph nodes.

**Figure 2:**
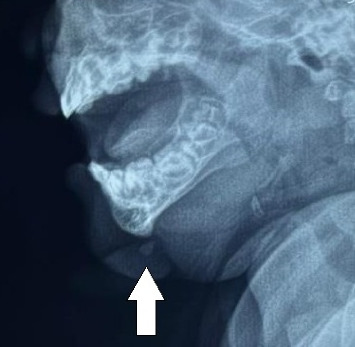
Plain Lateral Radiograph of the face showing a well-defined soft tissue opacity extending from the mental protuberance of the mandible anteroinferiorly with a focus of radiodensity similar to the tongue and other soft tissues within.

Her hemogram and serum alpha-fetoprotein were within normal limits (s.afp 0.40 u/mL; normal reference range < 6.05 units/mL). Plain lateral radiograph of the mandible showed a well-defined soft tissue opacity extending from the mental protuberance of the mandible anteroinferiorly with a focus of radiodensity within, as shown in Figure 2.

**Figure 3:**
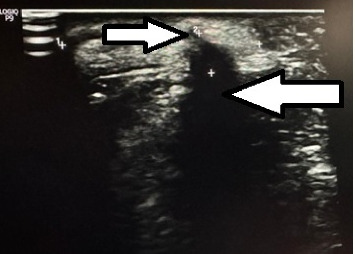
Ultrasound of the facial swelling showing a finger-like projection arising from the border of the mandible with a hyperechoic structure internally at the base (upper arrow) casting a posterior acoustic shadow (lower arrow).

Ultrasonogram (USG) of the swelling showed a finger-like projection arising from the border of the mandible with a hyperechoic structure internally at the base that was casting a posterior acoustic shadow and a surrounding hyperechoic soft tissue. Another small homogenously hyperechoic structure was noted adjacent to this, as shown in Figure 3.

**Figure 4:**
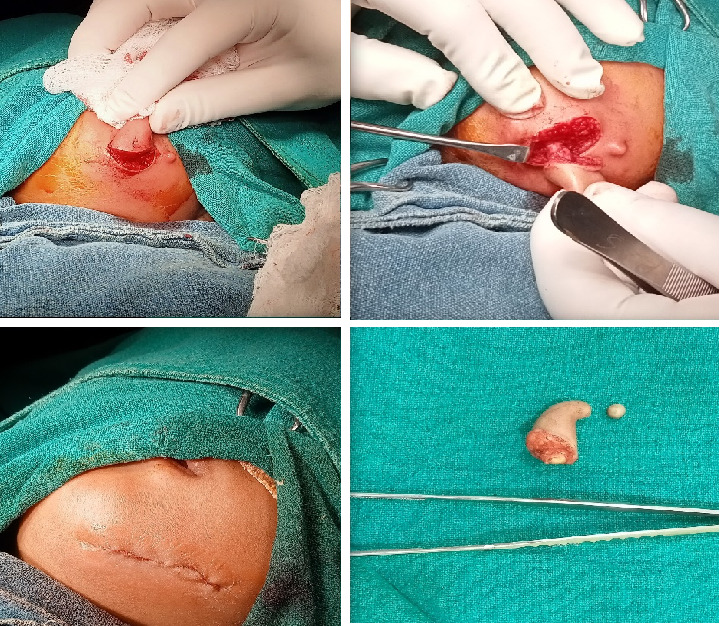
Intraoperative view of the facial teratoma.

A presumptive diagnosis of a faciomandibular teratoma with an adjacent skin tag was made and subsequently excised using an elliptical skin incision skirting around the swellings, as shown in Figure 4.

**Figure 5:**
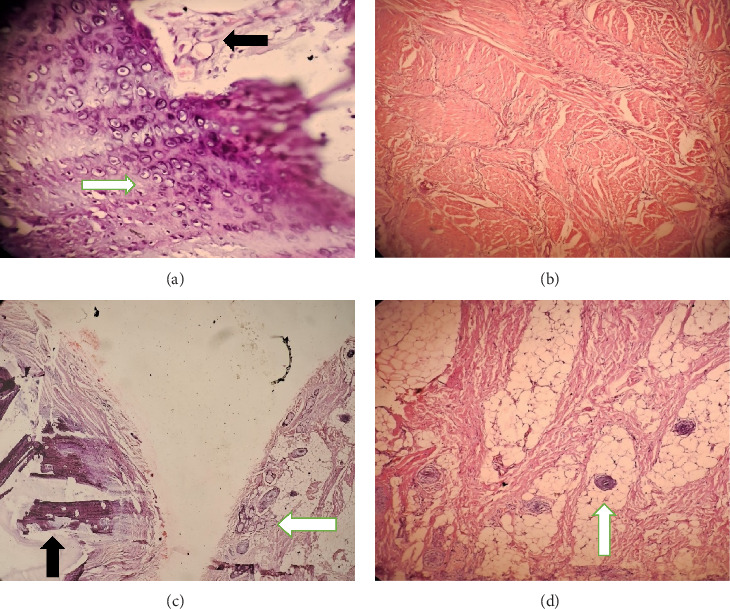
The histopathology of facial lesion stained with hematoxylin and eosin (H&E) stain: (a) 40× magnification H&E stain showing cartilage (white arrow) and bony elements (black arrow) at the base. (b) H&E stain 10× magnification shows thick muscle bundles in the deeper parts away from the skin surface. (c) H&E stain 10× magnification shows skin adnexal structures within the adipose tissue (black arrow) and bone (white arrow). (d) H &E 40× magnification shows skin adnexal structures deep within the adipose tissue (white arrow).

Intraoperatively, the swelling was found to be attached with the inferior border of mandible with a cartilaginous stalk that needed to be nibbled off. The histopathology of the specimen showed epidermis and dermis with hair follicles, sebaceous glands, adipose tissues, muscle bundles, and features of calcification without immature components, as shown in Figure 5. The findings confirmed the diagnosis of a mature teratoma.

Outpatient follow-up at 2 weeks showed healthy wound healing and at 3 and 6 months, respectively; she had no features of recurrence.

## 3. Discussion

Teratomas are well-defined tumors that are often considered foreign to the area of actual location and consist of variable tissue layers along with solid or cystic components and distinctive elements such as bone or cartilage. Teratomas are broadly classified into mature and immature types based on histology and further graded by Gonzalez Crussi according to the percentage of immature components [3].

The etiology of teratomas, by and large, is unknown, although various theories have been postulated of which congenital inclusion of germ layers into deeper tissues at points where developing somatic regions fail to completely fuse during embryogenesis seems a plausible explanation for congenital teratomas [4].

Congenital teratomas of the face have the potential to be detected antenatally on ultrasound or MRI imaging. However, a few patients may present in a more dramatic manner in the immediate postnatal period with an obstructive mass resulting in respiratory distress requiring an immediate intervention [5]. Fortunately, our patient did not have such untoward symptoms and sought treatment later in infancy with concerns mainly related to cosmesis.

Plain facial radiographs of these lesions may show calcification in about 16% cases, while modalities such as ultrasonography, CT scan, and MRI are usually advantageous to identify the tumor components [6].

The index case represents a very unique site of occurrence for a teratoma considering its mandibular location. Therefore, an extensive literature search was done that resulted in tabulation of cases of facial teratoma at varied locations since 1996, as shown in Table 1.

In most situations, complete surgical excision and subsequent reconstruction is the treatment of choice [2, 13, 14]. The foreseeable complications include neurological deficits and facial deformities that warrant the need for detailed parental counseling explaining the risk of recurrence and the likelihood of additional interventions. However, based on reports, the early diagnosis of recurrence mandates close clinical follow-ups and imaging, with serum AFP levels being unreliable in most situations [15].

## 4. Conclusion

Facial teratomas are uncommon in the pediatric population, and furthermore, mandibular location is even rarer and unique. An integrated approach that includes utilization of proper surgical technique considering the esthetic and functional outcomes is imperative.

## Figures and Tables

**Table 1:** Worldwide case reports of facial teratoma.

S. No	Author/year	Age	Location/size	AFP (ng/mL)	Imaging	Operation	Histology
1	Biglioli et al. [7], 1996 Denmark	4 months	Left cheek (5 cm)	N/A	Adipose with a carrot-shaped cone of muscular tissue	Enucleation	Mature teratoma

2	Anderson and David [8], 2003 Australia	1 day	Left temporal	N/A	N/A	Total excision	Teratoma

3	Isik et al. [9], 2011 Turkey	1 day	Right side of the face and cranium (24 cm)	Abnormal	Heterogeneous mass with cystic components with areas of calcification	Total excision	Mixed immature teratoma

4	Rai et al. [2], 2011 India	1 day	Upper right cheek (3.5 cm)	N/A	Osseous growth arising from right lateral wall of the orbit	Total excision	Mature teratoma

5	Ram et al. [10], 2012 India	10 months	Right upper cheek (6 cm)	N/A	N/A	Total excision	Mature teratoma

6	Kadlub et al. [1], 2014	5 cases	Cervicofacial teratoma (13.5 cm)	Normal	Unilocular cyst with solid component	Complete tumor excision	Mature teratoma
			Temporo-maxillo-facial teratoma (8.3 cm)	Normal	Unilocular cyst with heterogeneous mass	Subtotal excision	Mature teratoma
			Epignathus (9 cm)	Normal	Multiple cysts with heterogeneous mass	Total excision	Mature teratoma
			Epignathus (7.8 cm)	Normal	No cystic component with heterogeneous mass	Subtotal excision	Mature teratoma
			Temporo-fronto-preauricular (6.6 cm)	Normal	Multiple cysts with heterogeneous mass	Total excision	Mature teratoma

7	Alexander et al. [11], 2015 UK	Newborn	Temporal mass (6 cm)	N/A	N/A	Near total excision	Mature teratoma
		20 weeks	Right facial mass (18 cm)	N/A	N/A	Total excision	Immature teratoma

8	Kekre et al. [12], 2016 India	1 day	Left face	Normal	Solid and cystic areas of calcification	Complete excision	Mature tri-dermal teratoma

9	Yhoshu et al. [6], 2021 India	11 months	Left temporal mass (12 cm)	Normal	Mixed intensity mass, multicystic with areas of fat intensity	Complete excision	Mature cystic teratoma

Abbreviations: AFP = alpha-fetoprotein; N/A = not available.

## Data Availability

The data that support the findings of this study are openly available if requested to the author.
